# Isolated murine skeletal muscles utilize pyruvate over glucose for oxidation

**DOI:** 10.1007/s11306-022-01948-x

**Published:** 2022-12-08

**Authors:** Ram B. Khattri, Jason Puglise, Terence E. Ryan, Glenn A. Walter, Matthew E. Merritt, Elisabeth R. Barton

**Affiliations:** 1 Department of Applied Physiology and Kinesiology, College of Health & Human Performance, University of Florida, 124 Florida Gym, 1864 Stadium Road, Gainesville, FL 32611 USA; 2 Department of Biochemistry and Molecular Biology, University of Florida, Gainesville, USA; 3 Department of Physiology and Functional Genomics, University of Florida, Gainesville, USA; 4 Myology Institute, University of Florida, Gainesville, USA; 5 Center for Exercise Science, University of Florida, Gainesville, FL USA

**Keywords:** Glucose metabolism, Nuclear magnetic resonance (NMR), Skeletal muscle metabolism, Metabolic tracer, Substrate specificity

## Abstract

**Introduction:**

Fuel sources for skeletal muscle tissue include carbohydrates and fatty acids, and utilization depends upon fiber type, workload, and substrate availability. The use of isotopically labeled substrate tracers combined with nuclear magnetic resonance (NMR) enables a deeper examination of not only utilization of substrates by a given tissue, but also their contribution to tricarboxylic acid (TCA) cycle intermediates.

**Objectives:**

The goal of this study was to determine the differential utilization of substrates in isolated murine skeletal muscle, and to evaluate how isopotomer anlaysis provided insight into skeletal muscle metabolism.

**Methods:**

Isolated C57BL/6 mouse hind limb muscles were incubated in oxygenated solution containing uniformly labeled ^13^C_6_ glucose, ^13^C_3_ pyruvate, or ^13^C_2_ acetate at room temperature. Isotopomer analysis of ^13^C labeled glutamate was performed on pooled extracts of isolated *soleus* and *extensor digitorum longus* (EDL) muscles.

**Results:**

Pyruvate and acetate were more avidly consumed than glucose with resultant increases in glutamate labeling in both muscle groups. Glucose incubation resulted in glutamate labeling, but with high anaplerotic flux in contrast to the labeling by pyruvate. Muscle fiber type distinctions were evident by differences in lactate enrichment and extent of substrate oxidation.

**Conclusion:**

Isotope tracing experiments in isolated muscles reveal that pyruvate and acetate are avidly oxidized by isolated soleus and EDL muscles, whereas glucose labeling of glutamate is accompanied by high anaplerotic flux. We believe our results may set the stage for future examination of metabolic signatures of skeletal muscles from pre-clinical models of aging, type-2 diabetes and neuromuscular disease.

**Supplementary Information:**

The online version contains supplementary material available at 10.1007/s11306-022-01948-x.

## Introduction

Healthy human mass is 40% skeletal muscle. These skeletal muscles have important functions that include motion, support, protecting vital organs, thermogenesis, breathing, and consequently require large amounts of fuel (Velloso, 2008). Depending on different factors such as workload, transporter activity, cellular uptake, and substrate availability, these muscles can utilize glucose, mono-carboxylic acids, amino acids, ketones, or fatty acids as preferential fuel sources (Barton et al., 2010; Velloso, 2008). Because oxidative metabolism is also a key factor in the efficiency of substrate utilization, mitochondria are significant contributors to fuel selection of oxidation (Kuzmiak-Glancy & Willis, 2014).

The physiological management of substrate uptake by skeletal muscle is a complex process following the delivery of substrates through the circulation. To understand the muscle specific processes underlying substrate management, isolated muscles superfused in physiologic media can be used. In the absence of any circulatory system, the isolated muscles depend on diffusion to penetrate the muscle tissue and to distribute oxygen, nutrients, and other substrates for metabolism (Kjøbsted et al., 2021). While oxygen can freely pass through the muscle membrane, other substrates rely on transport proteins to enter the sarcoplasm and, in many cases, the mitochondrial matrix. Glucose uptake requires glucose transporter type 4 (GLUT4) activity (Chen et al., 2015; Lu et al., 2019; Otero et al., 2016). Similarly, pyruvate uptake is regulated by monocarboxylate transporters 1-4 (MCT1-4) (Halestrap, 2012), whereas acetate requires carnitine palmitoyltransferase I (CPT1) for its uptake into the mitochondria of skeletal muscles (Arduini & Zammit, 2017). Once these substrates are within the cell, they can be used for energy production or stored for later use, such as the conversion of glucose to storage as glycogen (or triglyceride) by means of phosphorylation (Bickel, 2004). Several elegant studies have mapped out substrate uptake and preferential fuel utilization using isolated rodent muscles under physiological conditions (Hansen et al., 1995; Ploug et al., 1987). For instance, glucose uptake is commonly assessed using 2-deoxyglucose (2DG), which competitively inhibits glucose-6-phosphate isomerase, trapping glucose-6-phosphate in the cytosol. While 2DG uptake measurements provide insight into the initial major step in glucose handling, simple uptake measures are often insufficient to assess the metabolic state of the muscle. As such, 2DG assays are often used as a proxy of glucose oxidation, yet they cannot directly report on glycolytic metabolism (lactate production) or flux through pyruvate dehydrogenase (PDH). Substrate isotope labeling with ^13^C provides greater insight into the fate of substrates, where metabolomic analysis can identify metabolites that accumulate a ^13^C label following the use of the labeled substrate (Quattrocelli et al., 2019). An important extension of isotope tracing by mass spectrometry (MS) is the employment of NMR, which has the ability to quantify metabolite production, but can also be used to measure the fate of metabolites in the tricarboxylic acid (TCA) cycle (Wiechert, 2001; Wiechert et al., 2001)**.** As precise atom mappings are known for the TCA cycle, substrate preference in skeletal muscle can be determined using specifically ^13^C enriched fuel sources. Entry of carbon into the TCA cycle occurs through either acetyl-CoA and/or by means of anaplerotic pathways (Walton et al., 2003), and taking advantage of isotopomer analysis of ^13^C NMR spectra, relative flux can be determined from carbon-13 spectra taken at single time points (Chatham et al., 1995; Ragavan et al., 2021). Glutamate is key to this analysis as it is available in ample amounts with well-resolved multiplets in conventional ^13^C 1D NMR. Further, its rapid exchange with α-ketoglutarate makes it a favorable candidate to track TCA cycle flux (Gorietti et al., 2015; Malloy et al., 1990). Glutamate peaks enriched in ^13^C labeling on specific carbons distinguish the extent of different metabolic pathways: labeling on C-4 occurs from anaplerosis, whereas labeling on C-3 occurs from oxidative glutamate production. Hence, the fractional enrichment in ^13^C labeled glutamate makes it possible to determine TCA cycle and anaplerotic fluxes, where C4/C3 serves as an index of anaplerosis (Malloy et al., 1990).

The ability of NMR to detect ^13^C enriched intermediary metabolites of TCA cycle that result by uniform utilization of ^13^C-enriched substrates and chemical selectivity nature of carbon-13 NMR makes it a valuable tool for delving into normal metabolism (Cohen, 1983) as well as disrupted metabolic homeostasis associated with diseases such as diabetes and nonalcoholic fatty liver diseases (Samuel & Shulman, 2018). However, few studies to date have use this technique to determine relative substrate preference in isolated murine muscles. Preclinical studies of muscle contractile function often employ ex vivo preparations of the *soleus* and/or *extensor digitorum longus* (EDL) muscles which are relatively easy to prepare and represent slow and fast fiber properties, respectively. Therefore, the current study sought to examine the utility of this preparation for understanding the metabolic fuel utilization in isolated resting mouse muscles at room temperature. ^13^C-labeling in both muscle types was performed using three fuels: glucose, pyruvate, and acetate, followed by NMR-based metabolomics analyses. Incubating ^13^C-labeled substrates in the isolated skeletal muscles makes it possible to examine TCA cycle flux and substrate selection by these muscles.

## Materials and methods

### Materials

All ^13^C labeled or unlabeled chemicals utilized in this study were purchased from commercial sources and used without further purification. [U-^13^C_6_] glucose and [U-^13^C_2_] acetate were purchased from Sigma Aldrich (St. Louis, MO). [U-^13^C_3_] pyruvate along with deuterated 4,4-dimethyl-4-silapentane-1-sulfonic acid (DSS) and deuterium oxide (D_2_O) were obtained from Cambridge Isotope Laboratories (Tewksbury, MA). Unlabeled buffer agents (mono-and bi-basic phosphates, sodium azide, sodium chloride (NaCl), potassium chloride (KCl), calcium chloride (CaCl_2_), magnesium sulfate (MgSO_4_), 4-(2-hydroxyethyl)-1-piperazineethanesulfonic acid (HEPES) buffer, unlabeled glucose, and ethylene diamine tetra-acetic acid (EDTA)) were also obtained from Sigma Aldrich, St Louis, MO, USA. Ringer’s solution ((in mM) 120 NaCl, 4.7 KCl, 2.5 CaCl_2_, 1.2KH_2_PO_4_, 1.2MgSO_4_, 25 4-(2-hydroxyethyl)-1-piperazineethanesulfonic acid, and 5.5 glucose) gas equilibrated with 95% O_2_ and 5% CO_2_ to pH 7.4 or minimum essential media (MEM) (Fisher) containing 292 mM L-glutamine, 52 mM L-isoleucine, 52 mM L-leucine, 15 mM L-methionine, 32 mM L-phenylalanine, 48 mM L-threonine, 10 mM L-tryptophan, 52 mM L-Tyrosine disodium salt dehydrate, and 46 mM valine, was used to support isolated muscles.

### Animal care and husbandry

All procedures were approved by the Institutional Animal Care and Use Committee of the University of Florida. Male C57BL/6 mice 16 ± 3 weeks old were housed at 22 °C with a 12-h light/dark cycle and free access to ad libitum chow diet food and water.

### Isolated muscle sample preparation

Mice were anesthetized (using a combination of xylazine (80 mg/kg) and ketamine (10 mg/kg)) to allow removal of *soleus* and *extensor digitorum longus* (EDL) muscles. Upon removal, muscles were incubated at 22 °C in Ringer/MEM solution gas equilibrated with 95/5% O_2_/CO_2_ with appropriate ^13^C labeled substrates in a perfusion chamber routinely used for isolated muscle mechanics for 30 min. These included the following: 5.5 mM [U-^13^C_6_] glucose; 5.5 mM [U-^13^C_3_] pyruvate, or 16.5 mM [^13^C_2_] labeled Na-acetate. Following incubation, muscles were quickly removed, blotted, and then rapidly frozen in liquid nitrogen for subsequent NMR analysis. N = 4 muscles were pooled into a single biological replicate of 30–50 mg tissue to afford detectable levels of substrates in the NMR analysis.

### NMR measurements

Perchloric acid (PCA) or acetonitrile:isopropanol:water (3:3:2) extractions were performed for all samples to isolate metabolites. The latter method was more efficient in sample recovery due to the reduced number of steps in the procedure but did not affect the proportion of metabolites. For PCA extraction, isolated muscle samples were homogenized with a FASTPREP-24 (MP Biomedicals, Solon, Ohio, USA) with 6% (v/v) ice cold PCA and centrifuged at 4 °C. The solid muscle portion was washed again with the 6% (v/v) ice cold PCA followed by centrifugation at 4 °C. The supernatant (combined) obtained was further neutralized with 5 M potassium hydroxide and centrifuged again. The resulting supernatants were then lyophilized (Thermo-Scientific, Dallas, USA). The pH of the dried powder was adjusted to 7.2 after dissolving it in 200 μL of ultra-pure water using 1 M sodium hydroxide and 1 M hydrochloric acid. The pH-adjusted solution was further centrifuged, the resulting supernatant was dried and the powder was used to prepare the NMR sample.

For acetonitrile:isopropanol:water extraction, homogenization of isolated muscle samples was carried out in 1 mL acetonitrile:isopropanol:water (3:3:2, v:v:v) ice cold mixture with a FASTPREP-24 (MP Biomedicals, Solon, Ohio, USA) and centrifuged at 4 °C in separate vials. Resultant supernatants were further lyophilized till dryness (Thermo-Scientific, Dallas, USA). The dried powder was further dissolved in 1 mL of Acetonitrile:Water (1:1, v:v) mixture, vortexed well for ~ 5 min. The resultant solution was further centrifuged, the supernatant obtained was further dried and the powder was used to prepare the NMR sample. The centrifugation speed for each step used was 13.2 K rpm.

Each NMR sample consisted of 50 mM phosphate buffer (pH 7), 2 mM EDTA, 0.02% of NaN_3_ with 0.5 mM of DSS as a standard internal reference in deuterated environment. ^1^H NMR spectra were taken at 25 °C using a 600 MHz Bruker Avance II Console equipped with a TCI CryoProbe that utilized Bruker Topspin 4 software (Bruker BioSpin Corporation, Billerica, MA, USA). The first slice of a NOESY pulse sequence (noesypr1d) was used to acquire proton NMR (Lohr et al., 2019; Osis et al., 2019; Ravanbakhsh et al., 2015). Fractional enrichment for glutamate, lactate and alanine were determined using ^13^C decoupling ON/OFF ^1^H proton spectra as well as 1D NOESY spectra. To determine enrichments, a standard zgig pulse sequence was adapted to allow ^13^C decoupling during the acquistion period (1.36 s) to remove the satellites. Total enrichment was measured by taking a ratio of the metabolite peak heights in the decoupling on/off experiments. NOESY spectra were collected with a 1 s relaxation delay (d1), and a 4 s acquisition time (at), in accordance with Chenomx recommendations for producing quantitative estimates of concentration. Using the Chenomx quantification and the fractional enrichments, a final concentration of the metabolites was calculated. Conventional ^1^H decoupled ^13^C spectra were acquired using a 600 MHz Agilent with a specially designed 1.5 mm superconducting (HTS) probe at 30 °C (J N Thomas et al., 2022). All the parameters utilized to acquire these NMR spectra are shown in Supporting Table ST1.

Metabolites were assigned with the help of previous publications (Khattri et al., 2021b; Yoshioka et al., 2002), biological magnetic resonance bank (BMRB) (Ulrich et al., 2008), and 1D proton spectra acquired for this study.

### NMR spectra processing

Proton spectra were zero filled to 64 K, whereas carbon spectra were zero filled to 128 k data points before Fourier transformation, with exponential line broadening of 0.5 Hz using MestReNova 11.0.0–17,609 (Mestrelab Research, S.L., and Santiago de Compostela, Spain). DSS singlet resonance at 0 ppm was used to calibrate proton spectra. The carbon spectra were calibrated to a taurine singlet resonance at 48.4 ppm. Both proton and carbon spectra were baseline corrected with Whittaker-Smoother or spline as required. For carbon spectra, glutamate resonances were fitted with a mixed Gaussian/Lorentzian lineshape and integrals were measured. Integrals values were utilized to calculate peak multiplet ratios of isotopomers. Metabolite concentrations were determined using Chenomx NMR Suite 8.2 (Chenomx, Inc., Edmonton, Alberta, Canada), using ^1^H 13C decoupling ON spectra with respect to DSS peak at 0.00 ppm (0.5 mM added concentration). The concentration values thus obtained were further normalized to tissue mass while maintaining 35 µL of NMR sample (for both soleus and EDL muscles) to enable comparisons across each replicate of pooled samples.

### Determination of [1,2 ^13^C_2_] enriched acetyl-CoA and anaplerotic flux

A non-steady-state analysis was performed to determine the enrichment in acetyl-CoA (Fc3) and anaplerotic flux (Y), using total ^13^C enrichment in C3 and C4 carbon atoms of glutamate (Malloy et al., 1990). The following equations were used:

1 $$ Fc3 = \, \left( {{\text{C4Q}}} \right)\left( {{\text{C4}}/{\text{C3}}} \right) $$

2 $$ Y = {\text{ C4}}/{\text{C3}} $$

Here, C4Q represents the total area of the quartet (doublet of doublets) resonances for glutamate carbon C4 resulting from the C4_345_ isotopomer. C4 is the total area of the 4-carbon resonance and C3 is the total area of the 3-carbon resonance of glutamate. Carbons C4 and C3 have essentially idenitical signal dependencies on the nuclear Overhauser effect and the same T_1_, therefore accurate C4/C3 ratios are easily collected.

### Statistical analysis

Unpaired Student’s *t*-test (for two groups), ordinary one-way ANOVA with Tukey’s post hoc analysis (for comparison of more than two groups), and two way ANOVA with Sidak’s multiple comparisons test were used to determine the significant levels in concentration differences for metabolites. A *p*-value < 0.05 was considered statistically significant.

## Results

NMR can be used to measure pool sizes of metabolites as well as metabolic flux. In this study, conventional ^13^C one-dimensional NMR was utilized to measure a shift in metabolic fuel utilization for isolated murine muscles. EDL and soleus muscles were incubated with oxygenated minimal essential media (MEM) or Ringers solution containing uniformly labeled [^13^C_6_] glucose or [^13^C_3_] pyruvate or [U-^13^C_2_] acetate at room temperature and were analyzed by NMR. Incubation in [U-^13^C_2_] acetate was utilized to confirm intact metabolism.

### Incubation media alters abundance of branched chain amino acids but not fractional enrichment or anaplerotic flux

To determine if media conditions would alter the fate of labeled substrates, two different conditions were examined: (i) amino acid free buffer (Ringer’s solution) which may cause a net negative protein balance that might result in muscle protein degradation to provide amino acids for metabolic flux and (ii) MEM media, which is replete with amino acids. As anticipated, greater branched chain amino acid (BCAA) abundance (leucine, isoleucine, and valine; over seven-fold greater in both EDL and soleus muscles) was detected in MEM (indicated by red letters in Supporting Table ST2), but amino acids contributing to glycolysis and TCA were unaffected. Further, fractional enrichment (%) for lactate and alanine are similar for both MEM and Ringer’s (Supporting Table ST3). The fraction of [1,2 ^13^C_2_] enriched acetyl-CoA (Fc3) produced by [U-^13^C_3_] pyruvate treatment was 2.5-fold lower in MEM media for EDL muscle as compared to Ringer’s solution (Supporting Table ST4). The Fc3 value by [U-^13^C_6_] glucose treatment was undetectable due to absence of C4Q quartets in C4 carbon of glutamate. However, the anaplerotic flux (C4/C3) values did not differ between MEM and Ringer’s solution.

### Substrate-dependent oxidation in isolated muscles

#### Pyruvate is a superior substrate

**Fig. 1:**
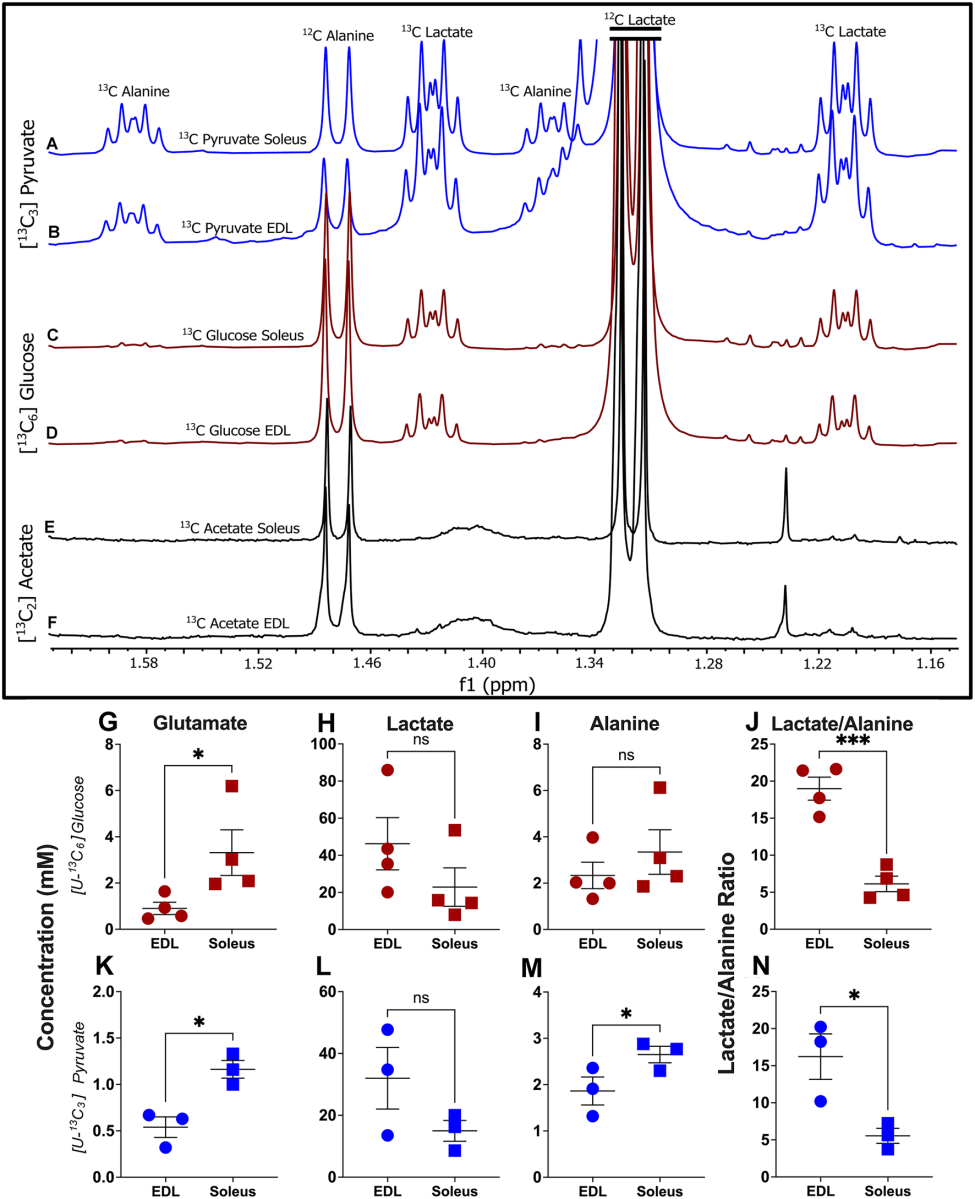
Representative ^1^H 1D NOESY spectra from EDL and soleus extracts treated with [U-^13^C_6_] glucose, [U-^13^C_3_] pyruvate, and [^13^C_2_] acetate, separately (Fig. 2A–F). Greater lactate enrichment and pronounced alanine enhancement is observed for [U-^13^C_3_] pyruvate treated extracts (Fig. 2A, B). Modest lactate enrichment and nominal alanine enrichment is seen for [U-^13^C_6_] glucose treated extracts (Fig. 2C, D). No lactate as well as alanine enrichment observed for [^13^C_2_] Na-acetate treated extracts by 1D ^1^H NMR (Fig. 2E, F). Figure 2G–N are the scatter dot plots showing the concentrations of key TCA and glycolytic metabolites for soleus and EDL tissue samples incubated with ^13^C labeled [U-^13^C_6_] glucose (n = 4) and [U-^13^C_3_] pyruvate (n = 3). Concentrations were determined using Chenomx NMR Suite 8.2 software, with ^13^C decoupling ON 1D ^1^H spectra. Each NMR sample is the extract of either four EDL or soleus muscles. Four biological replicates were used for [U-^13^C_6_] glucose treated samples, and three biological replicates were used for [U-^13^C_3_] pyruvate treated samples. Each biological replicate was a pool of 4 individual muscles. Statistical significance was determined using unpaired t-tests via GraphPad Prism with p < 0.05 considered significant; p < 0.05 is denoted with *p < 0.01 is denoted with **p < 0.001 is denoted with ***

Irrespective of muscle, [U-^13^C_6_] glucose labelled samples showed modest lactate enrichment (~ 6–16%) along with minimal alanine enrichment (21–28%), indicating larger involvement of other carbon sources besides [^13^C_6_] glucose. In contrast, samples labelled with [U-^13^C_3_] pyruvate showed greater lactate (13–32%) and alanine enrichment (59–67%), demonstrating that pyruvate is a superior substrate for resting muscles in isolated preparations (Fig. 1A–D). Importantly, we did not detect lactate or alanine enrichment in muscles labeled with [^13^C_2_] acetate (Fig. 1E, F). Fractional enrichments (%) with their respective standard deviation are shown in Table 1. Not surprisingly, lactate enrichment was significantly different in EDL and soleus muscles within each substrate; however, no difference was observed for alanine enrichment.

**Table 1:** Fractional enrichment (%) for lactate and alanine in muscle extracts

	Fractional enrichment (%)			
Substrate	[U-^13^C_6_] glucose		[U-^13^C_3_] pyruvate	
Metabolite	Alanine (n = 4)	Lactate (n = 4)	Alanine (n = 3)	Lactate (n = 3)
EDL	22.15 ± 8.89^###^	5.57 ± 0.67**,^#^	66.45 ± 5.91	13.80 ± 4.19
Soleus	27.64 ± 8.03^###^	15.74 ± 1.68	63.69 ± 6.13	25.06 ± 3.44**,^#^

Results are stated as mean ± S.D. Enrichment was calculated using ^13^C decoupling ON/OFF ^1^H proton spectra as well as ^1^H 1D NOESY spectra. Each NMR sample is the extract of either four EDL or soleus muscles. Four biological replicates were used for [U-^13^C_6_] glucose treated samples, and three biological replicates were used for [U-^13^C_3_] pyruvate treated samples. *N.D* not determined. Each biological replicate was a pool of 4 individual muscles. Statistical significance was determined using two way ANOVA with Sidak’s multiple comparisons. *, differences between muscles in the same substrate. #, differences between substrates in the same muscle

*/#p < 0.05; **/## p < 0.01; and ***/###p < 0.001

#### Metabolism is consistent with fiber type profiles of EDL and soleus muscles

Proton (^1^H) NMR can be utilized to quantify metabolites with high reproducibility (Khattri et al., 2021a; Lohr et al., 2019). However, utilization of ^13^C-labelled substrates may complicate the accuracy of metabolite quantification because of the appearance of ^13^C satellite peaks for labeled metabolites. To overcome this issue, we utilized ^13^C-decoupled 1D ^1^H spectra to determine concentration of metabolites. Comparisons of metabolite concentrations were performed between soleus (60% myosin heavy chain I) and EDL (> 90% myosin heavy chain II) muscles. Soleus muscles showed significantly higher amounts of glutamate and alanine in both [U-^13^C_6_] glucose and [U-^13^C_3_] pyruvate treated samples (Fig. 1G–N) compared to EDL muscles, which is consistent with the oxidative nature of slow myofibers. Similarly, lactate abundance was ~ threefold greater in fast-twitch EDL muscle compared to soleus. The lactate to alanine ratio supported the predominance of glycolytic metabolism in EDL compared to the soleus muscle samples (p = 0.05–0.001).

#### Concentrations of BCAA, TCA, and glycolytic metabolites indicate no change in energy demand by different substrates

No differences in the concentrations of glutamate, lactate, or alanine or in the lactate to alanine ratio were found in soleus and EDL muscles regardless of the ^13^C substrate used (Supporting Fig. S1). Further, concentrations of branched chain amino acids were not altered in the substrate conditions (Supporting Fig. S2) as well as in two muscle types (Supporting Fig. S3).

#### Pyruvate and acetate are avidly oxidized by isolated muscles

**Fig. 2:**
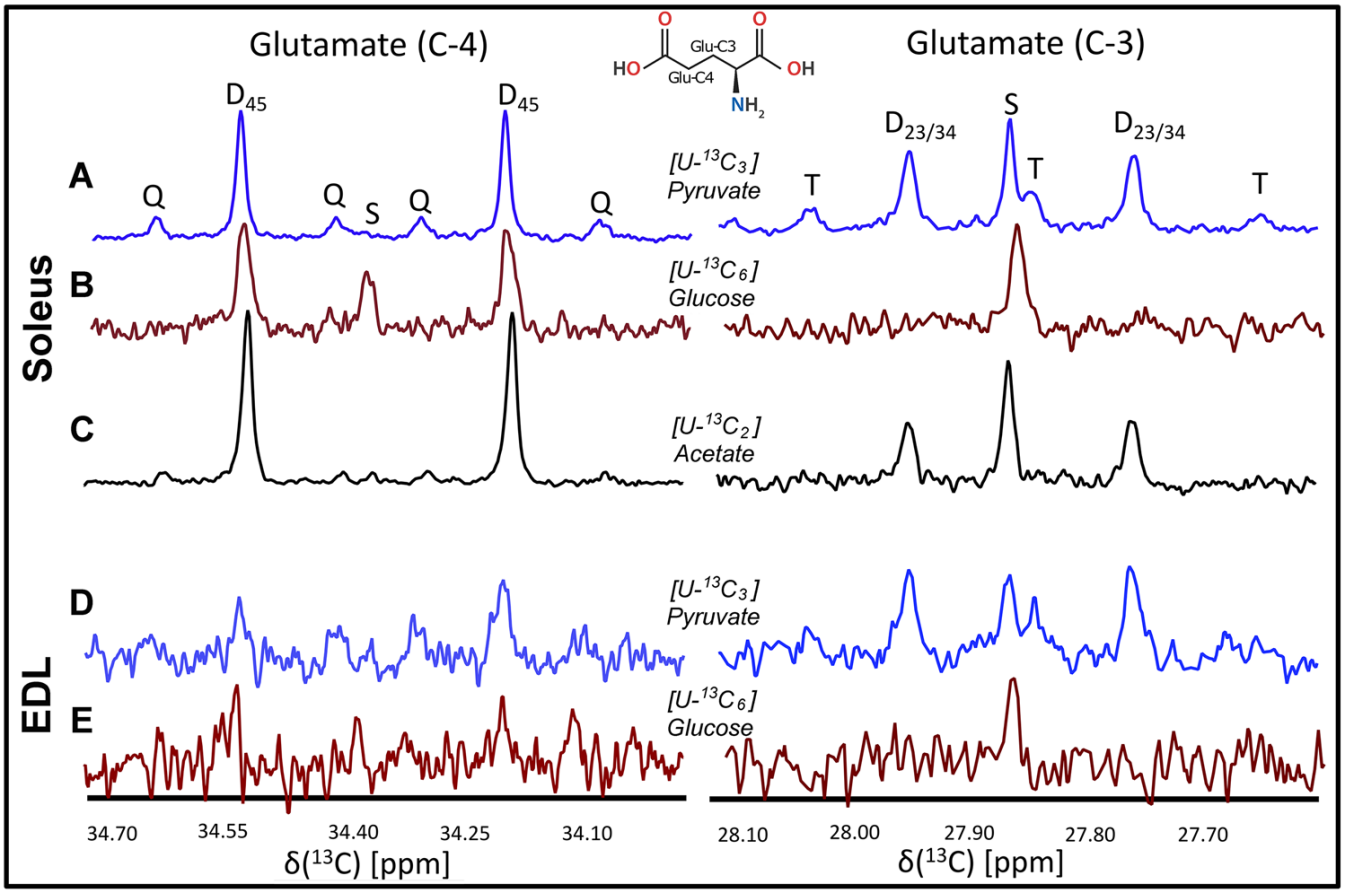
Representative ^13^C spectra (^1^H decoupled) of PCA extracts obtained from the incubation of three different types of ^13^C labeled substrates in different tissue samples for 30 min in MEM/ringer media, separately. Glutamate C3 and C4 resonances are shown. Top three spectra (A, B, & C) are for soleus tissue samples infused with [U-^13^C_3_] pyruvate, [U-^13^C_6_] glucose, and [U-^13^C_2_] acetate, respectively. Bottom two spectra (D & E) are for EDL tissue samples infused with [U-^13^C_3_] pyruvate and [U-^13^C_6_] glucose, respectively. Different labelling patterns for glutamate C4 and C3 carbons are presented. Visible multiplets such as ‘D’, ‘T’, and ‘Q’ represent respective doublet, C234 triplet, and C345 quartet, respectively. ‘S’ represents C4 or C3 singlet. Glu is glutamate

Isotopomer analysis of glutamate with 1D ^13^C conventional NMR indicated that [U-^13^C_3_] pyruvate and [U-^13^C_2_] acetate were oxidized to a higher degree than glucose (Fig. 2). Presence of glutamate C4D45 doublets and C4Q345 quartets in [U-^13^C_3_] pyruvate treated samples demonstrated that [U-^13^C_3_] pyruvate was extensively oxidized in isolated muscles (Fig. 2A, D). The Fc3 value was 41 ± 3% in [U-^13^C_3_] pyruvate treated soleus and 46 ± 21% in [U-^13^C_3_] pyruvate treated EDL muscles (Table 2). [U-^13^C_2_] acetate labeled glutamate in soleus muscles (Fig. 2C) with an Fc3 value of 40.1%. Poor oxidation of [U-^13^C_6_] glucose was further supported by the C4 signature of glutamate, which lacked the C4Q quartet in both soleus and EDL samples, and therefore, Fc3 was not possible to measure (Fig. 2B, E). However, for all three ^13^C labeled substrate treatment groups, soleus samples showed higher oxidation compared to EDL samples, consistent with their higher density of mitochondria (Isaeva et al., 2005).

**Table 2:** Non-steady-state analysis of fractional enrichment and anapleurotic flux

	Fractional enrichment Fc3			Anaplerotic flux C4/C3		
Substrate	[U-^13^C_6_] glucose (n = 4)	[U-^13^C_3_] pyruvate (n = 3)	[U-^13^C_2_] acetate (n = 1)	[U-^13^C_6_] glucose (n = 4)	[U-^13^C_3_] pyruvate (n = 3)	[U-^13^C_2_] acetate (n = 1)
EDL	ND	0.46 ± 0.21	ND	1.43 ± 0.19***	0.90 ± 0.10	ND
Soleus	ND	0.41 ± 0.03	0.40	2.59 ± 0.10***,^###^	1.23 ± 0.27	4.68

Results are stated as mean ± S.D. Fc3 represents the fractional enrichment of [1,2^13^C_2_] acetyl-CoA, whereas C4/C3 is the anaplerotic flux. Each NMR sample is the extract of either four EDL or soleus muscles. Four biological replicates were used for [U- ^13^C_6_] glucose treated samples, and three biological replicates were used for [U-^13^C_3_] pyruvate treated samples. *N.D*  not determined. Each biological replicate was a pool of 4 individual muscles. Statistical significance was determined using two way ANOVA with Sidak’s multiple comparisons. *, differences between muscles in the same substrate. #, differences between substrates in the same muscle

*/#p < 0.05; **/##p < 0.01; and ***/###p < 0.001

High anaplerotic flux (C4/C3, Table 2) was observed for [U-^13^C_6_] glucose treated samples. The existence of intense glutamate C-3 singlets is indicative of high anaplerotic flux via succinate or other cataplerotic pathways (Ragavan et al., 2021), which resulted in reduction of ^13^C enrichment in the interior carbons of glutamate. The anaplerotic flux (C4/C3) for oxidative soleus muscle was higher than the glycolytic EDL muscle (2.59 ± 0.10 vs 1.43 ± 0.19, *p* < 0.001, Table 2). Anaplerosis was also found for both muscle types treated with [U-^13^C_3_] pyruvate (soleus: 1.23 ± 0.27; EDL: 1.09 ± 0.10) relative to TCA cycle turnover, but it was comparatively smaller than in the glucose treated samples (Table 2). This suggests that exogenous pyruvate is a superior substrate of oxidation compared with exogenous glucose in isolated muscles. In regard to fatty acid utilization, existence of a large C4D45 doublet (with C4/C3 value = 4.68, Table 2) for glutamate C4 in [U-^13^C_2_] acetate treated soleus muscles indicates that acetate was also utilizing anaplerotic flux to provide TCA cycle intermediates.

### Preincubation with pyruvate alters oxidation and anaplerotic flux

**Fig. 3:**
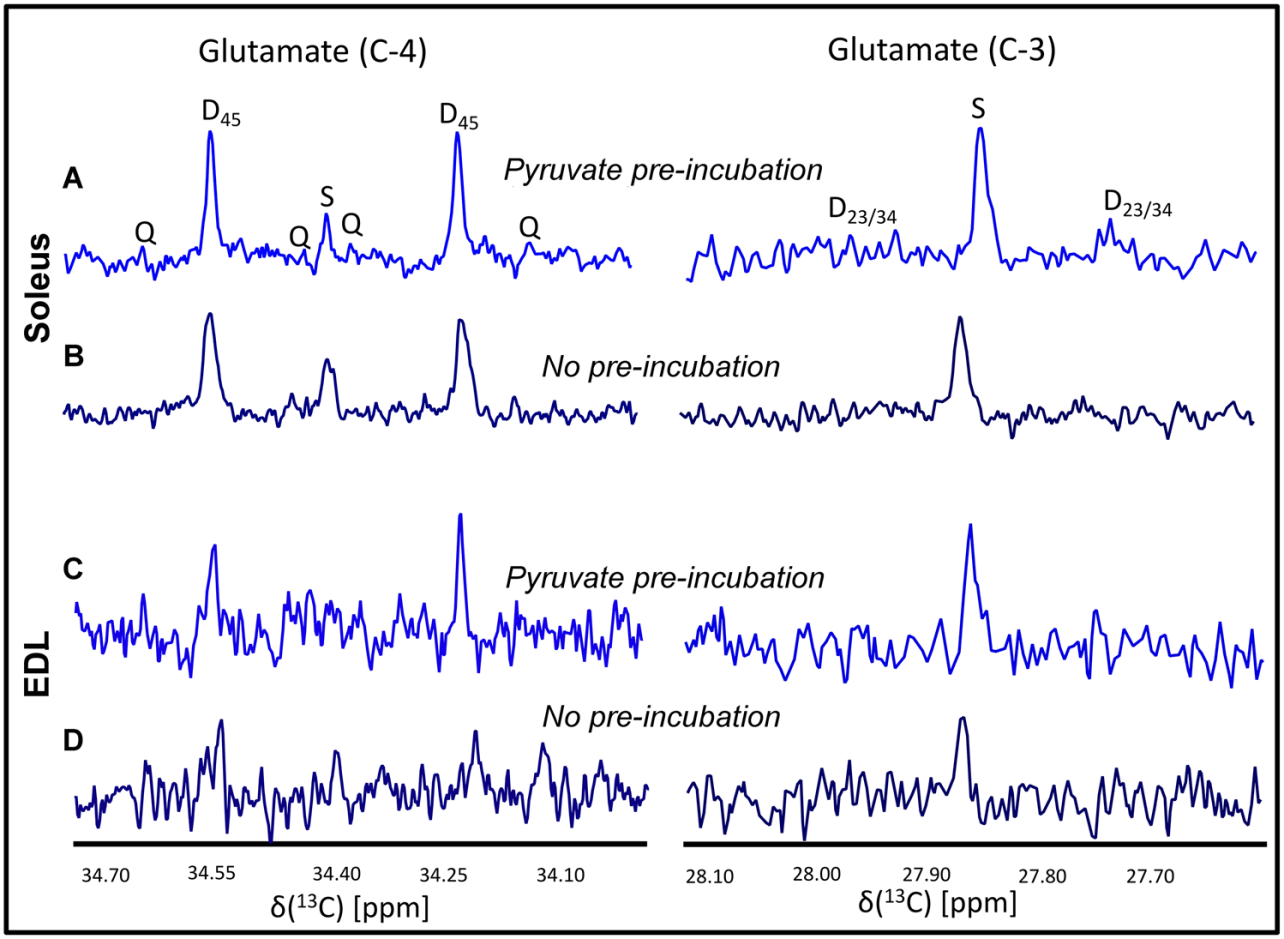
Representative ^13^C spectra (^1^H decoupled) of isolated soleus (A and B) and EDL (C and D) muscle extracts with and without pyruvate pre-incubation followed by incubation with [U-^13^C_6_] glucose for 30 min in MEM/ringer media. Glutamate C3 and C4 resonances are shown. Different labelling patterns for glutamate C4 and C3 carbons are presented. ‘S’ represents C4 or C3 singlet. Visible multiplets ‘D’, and ‘Q ‘ represent doublet and C345 quartet, respectively

PDH plays an important role in glucose/fatty acid utilization, and therefore, in glucose oxidation (Small et al., 2018). Previous studies have demonstrated that pre-incubation with pyruvate helps to elevate PDH activity and thus, increases glucose oxidation (Small et al., 2018). To determine if the minimal glucose oxidation observed in the above measurements was limited by PDH activity, EDL and soleus muscles were first pre-incubated with 2 mM unlabeled pyruvate in MEM for 30 min at room temperature. These samples were further incubated with 5.5 mM [U-^13^C_6_] glucose for another 30 min. Pre-incubation with pyruvate slightly elevated the oxidation of [U-^13^C_6_] glucose, especially in isolated soleus muscles as demonstrated by the presence of C4Q345 quartets in C4-carbon of glutamate, making it possible to measure *Fc3* values (*Fc3* = 0.245). On the other hand, little change was observed for isolated EDL muscles (Fig. 3). Significantly higher anaplerotic flux was observed for pyruvate pre-incubated samples (Supporting Table ST5) for both muscle types (2.5-and 1.5-fold increase in EDL and soleus muscles, respectively). Therefore, pre-incubation with pyruvate increased both glucose oxidation and anaplerotic flux to provide TCA cycle intermediates.

### Pyruvate labels more TCA cycle metabolites than glucose

**Fig. 4:**
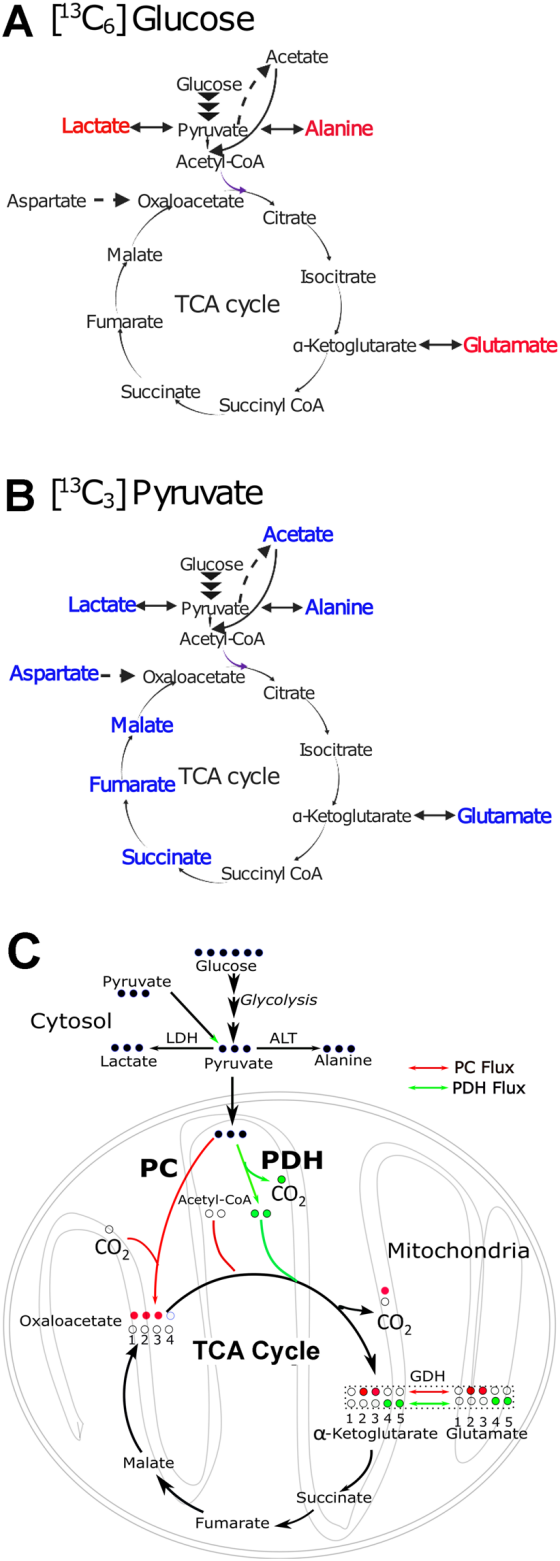
Incorporation of ^13^C labelling in different metabolites of glycolysis and Tricarboxylic acid (TCA) pathways by A) [U-^13^C_6_] glucose and B) [U-^13^C_3_] pyruvate in isolated EDL and soleus muscles, identified with the aid of 1D conventional ^13^C NMR spectra. Red letters: incorporation of ^13^C labelling by [U-^13^C_6_] glucose, and blue letters: incorporation of ^13^C labelling by [U-^13^C_3_] pyruvate. C. Possible metabolic pathway of [U-^13^C_6_] glucose, [U-^13^C_3_] pyruvate, and [U-^13^C_2_] acetate shown for isolated muscles via first turn of TCA cycle. Red, blue, and green circles represent the ^13^C labeled carbon backbone in the metabolites coming through PC and PDH flux, respectively. White circles represent unlabeled carbon backbone

1D conventional ^13^C NMR spectra enabled the detection of C-13 labels in TCA intermediates. [U-^13^C_6_] glucose was found to label only alanine, lactate and glutamate in EDL and soleus muscles (Fig. 4A). In contrast, [U-^13^C_3_] pyruvate labeled the majority of TCA cycle metabolites in both EDL and soleus muscles (Fig. 4B). The large number of ^13^C labelled metabolites for the [U-^13^C_3_] pyruvate is a demonstration of its superior oxidation by the isolated muscles. Of note were distinctions in the multiplet patterns created by [U-^13^C_3_] pyruvate and [U-^13^C_6_] glucose, which are indicative of the complexity of intermediary metabolites contributing to the TCA cycle (Cappel et al., 2019; Steiner et al., 2021). The limitations of the ^13^C NMR sensitivity combined with the low oxidation of [U-^13^C_6_] glucose prevented potential detection of the extent of labeling by this substrate.

### PDH is more active than pyruvate carboxylase (PC) for all ^13^C-labelled substrates

All three substrates labeled C-4 and C-5 carbons of α-ketoglutarate in the first turn of the TCA cycle via pyruvate dehydrogenase (PDH) flux. [U-^13^C_6_] glucose and [U-^13^C_3_] pyruvate also labeled C-2 and C-3 carbons of the α-ketoglutarate via pyruvate carboxylase (PC) flux. With the subsequent turns of TCA cycle, interior carbons of the α-ketoglutarate were labelled. Since α-ketoglutarate is in rapid exchange with glutamate via glutamate dehydrogenase activity, the carbons of glutamate also were labelled (Fig. 4C). After subsequent TCA cycles, a quartet C4Q345 was observed for the glutamate C-4 carbon signal with both [U-^13^C_3_] pyruvate and [U-^13^C_2_] acetate, but not with [U-^13^C_6_] glucose. This is an indication of poor oxidation of the [U-^13^C_6_] glucose. The doublet for C3 resonance of glutamate as a result of J23 coupling was observed in both soleus and EDL samples treated with [U-^13^C_3_] pyruvate and [U-^13^C_2_] acetate, indicating favorable PC flux (Fig. 2A, C, D). However, the doublet was not clearly visible in [U-^13^C_6_] glucose treated soleus (Fig. 2B) or EDL (Fig. 2E) samples, indicating poor oxidation of the [U-^13^C_6_] glucose in isolated muscle samples. The difference in multiplet patterns of glutamate obtained with ^13^C-NMR establish a platform to monitor different fluxes that participate in central energy metabolism.

## Discussion

NMR had been used in tracer-based studies for more than fifty years, but as sensitivity has improved with technological advances, the ability to detect metabolites with minimal tissue is possible (Saborano et al., 2019). In this study, we employed ^13^C tracer-based experiments to evaluate metabolic fuel utilization by isolated and incubated soleus and EDL muscles. With the combination of ^1^H and ^13^C NMR, we found that [U-^13^C_3_] pyruvate and [U-^13^C_2_] acetate were oxidized avidly by both soleus and EDL muscles compared to [U-^13^C_6_] glucose. However, soleus were more oxidative than EDLs, with significantly higher glutamate labeling. More multiplets were observed in C4-carbon of glutamate (including C345 quartets) for soleus compared to EDLs with all three-13C labeled substrates, substantiating its oxidative nature. Taken together, the fate of each labeled substrate could be traced from cell entry to utilization with as little as 30 mg tissue.

Soleus and EDL muscles are some of the best and most utilized examples of slow- and fast twitch muscles in mice. Murine soleus muscle is a representative slow-twitch type 1 fibers, whereas EDL muscle contains mostly fast-twitch type 2 fibers (Barclay & Weber, 2004). As anticipated, EDL muscles produced more lactate, and thus can be considered more glycolytic compared to soleus with all three ^13^C labelled substrates used in this study (Paré et al., 2017). On the other hand, soleus muscle produced more glutamate and alanine, showing its oxidative nature. Skeletal muscles are considered as one of the primary sources for BCAA catabolism (Khattri et al., 2021a). However, BCAA were not altered in either isolated muscle type with any of the ^13^C labeled substrates in our study, an indication of no change in energy demand by these substrates.

The position of carbon labeling represents the specific pathways used by substrates entering the TCA cycle. Here, we utilized all three substrates to determine relative flux through PDH and PC. Thirty minutes of incubation of ^13^C-substrates with isolated muscle is sufficient time to reach steady state (Ragavan et al., 2021), but there is a requirement for oxygen consumption measurements, which were not performed in this study. Instead, we took advantage of a non-steady state approach to monitor rapid substrate selection using peak ratios of glutamate multiplets (Malloy et al., 1990).

To date we found no literature that compares the oxidation of both glucose and pyruvate by isolated and incubated skeletal muscles. However, Jessen et al. reported minimal oxidation of glucose compared to pyruvate in oxidative pathways by perfused isolated hearts (Cobert et al., 2012). In spite of differences in cardiac and skeletal muscle metabolism, we also found elevated pyruvate oxidation by both soleus and EDL muscles. Presence of a C345 quartet in C4-carbon of glutamate in 1D ^13^C conventional NMR of pyruvate treated muscle extracts also support the elevated oxidation of pyruvate. The C345 quartet in C4 carbon of glutamate was absent in glucose treated muscle extracts, indicating that isolated muscles incubated with [U-^13^C_6_] glucose utilized endogenous substrates (that include glycogen or lipids) as fuels to carry out oxidative metabolism (Cobert et al., 2012; Liedtke et al., 1978; Liu et al., 2002; Neely & Grotyohann, 1984). The anaplerotic flux (C4/C3) values also support these statements, which was comparatively higher for [U-^13^C_6_] glucose treated muscles.

The mechanisms underlying ineffective utilization [U-^13^C_6_] glucose by these isolated muscles were not pursued, although the most plausible explanations include hypo-activity of glucose transporter type 4 (GLUT4), or utilization of endogenous glycogen. Incubation conditions, which include low temperature (25 °C), no stimulation, and no insulin, could all contribute to reduced glucose uptake as shown previously (Kjøbsted et al., 2021; Orme & Kelly, 1977; SHIPP et al., 1965; Wasserman et al., 2011). On the other hand, pyruvate is an attractive fuel source (Peltz et al., 2005a, 2005b). With the aid of monocarboxylate transporters, [U-^13^C_3_] pyruvate can easily pass through the cell membrane and enter the TCA cycle as acetyl-CoA by pyruvate dehydrogenase activity (Cobert et al., 2012; Halestrap, 2012). According to a previous study, pyruvate can act as an anaplerotic substrate and replenish TCA cycle intermediates with the help of pyruvate carboxylase activity (Panchal et al., 2000), leading to its higher oxidation in our current experiments. [U-^13^C_2_] acetate was also avidly oxidized by soleus muscles. The presence of a C345 quartet in C4 carbon of glutamate confirms its superior oxidation over [U-^13^C_6_] glucose. CPT1 can carry acetate through cell membranes to enter into the TCA cycle to form of acetyl-CoA (Arduini & Zammit, 2017). However, acetate incubation was accompanied by large anaplerotic fluxes, implying involvement of endogenous fuel sources to replenish TCA cycle metabolites. Together, the comparison of different substrate utilization in fast and slow skeletal muscles illustrates the multiple pathways invoked to utilize these fuels.

There are several limitations associated with this study. First, we needed to combine four muscles into a single biological replicate to achieve acceptable signal-to-noise in NMR spectra, particularly for conventional ^13^C NMR as compared to ^1^H NMR. Even with pooled samples, the time required to collect conventional ^13^C NMR spectra was extensive, in some cases more than 24 h. Second, the reported metabolite concentrations were calculated from ^13^C-decoupled 1D ^1^H spectra in order to suppress the ^13^C-satellite signals that can arise from use of ^13^C labeled substrates, and may impair accurate calculations. Third, we consider that the major portion of the substrates oxidized by the isolated muscles are coming from the exogenously added ^13^C labeled substrates. This ^13^C MRS method is incapable of directly measuring the oxidation of unlabeled endogenous fuel source present in the muscles. With the improvement in current technologies, additional studies should be conducted to refine our knowledge of the fuel preference and metabolome by these isolated and incubated small-sized skeletal muscles.

In conclusion, in vitro isotope tracing experiments reveals that pyruvate and acetate are avidly oxidized by isolated soleus and EDL muscles at room temperature, whereas glucose labels glutamate but with quite high anaplerotic flux. The use of [U-^13^C_3_] pyruvate may serve as a more efficient strategy to probe metabolism with stable isotopes bypassing the regulation of glucose uptake and subsequent utilization. We believe our results help to set the stage for future studies combining muscle function testing along with the broad evaluation of metabolic/anatomical signatures of skeletal muscles from pre-clinical models of aging, type-2 diabetes and neuromuscular disease.

## Supplementary Information

Below is the link to the electronic supplementary material.

Supplementary file1 (DOCX 266 KB)

## Data Availability

All data related to this study are included in the article and supporting elements. Raw Data is available via the Metabolomics Workbench. http://dev.metabolomicsworkbench.org:22222/data/DRCCMetadata.php?Mode=Study&StudyID=ST002356&Access=ThyQ3386. The DOI for this project (PR001513) is: 10.21228/M8471X.

## Abbreviations

2DG: 2-Deoxyglucose
DSS: 4,4-Dimethyl-4-silapentane-1-sulfonic acid,
BCAA: Branched chain amino acid
CPT1: Carnitine palmitoyltransferase I
D_2_O: Deuterium oxide
EDL: Extensor digitorum longus
GLUT4: Glucose transporter type 4
MS: Mass spectrometry
MEM: Minimal essential media
MCT1-4: Monocarboxylate transporters 1–4
NMR: Nuclear magnetic resonance
PCA: Perchloric acid
PC: Pyruvate carboxylase
PDH: Pyruvate dehydrogenase
TCA: Tricarboxylic acid
