# Supplementary material for: Isolated murine skeletal muscles utilize pyruvate over glucose for oxidation
Source: Metabolomics. 2022 Dec 8;18(12):105. doi: 10.1007/s11306-022-01948-x (PMC9732067; doi:10.1007/s11306-022-01948-x)
Supplement: Supplementary file 1 — Supplementary file1 (DOCX 266 KB) [file 11306_2022_1948_MOESM1_ESM.docx]

**SUPPORTING INFORMATION**

**Isolated murine skeletal muscles utilize pyruvate over glucose for oxidation**

Ram B. Khattri^1^, Jason Puglise^1^, Terence E. Ryan^1,4,5^, Walter, A. Glenn^3,4^, Matthew E. Merritt^2^, Elisabeth Barton^1,4,5^

^1^Department of Applied Physiology and Kinesiology;

^2^Department of Biochemistry and Molecular Biology;

^3^Department of Physiology and Functional Genomics;

^4^Myology Institute;

^5^Center for Exercise Science, University of Florida, Gainesville, FL, USA

**Key words:** Glucose metabolism, nuclear magnetic resonance (NMR), skeletal muscle metabolism, metabolic tracer, substrate specificity

**Running Title:** C-13 isopotomer analysis of skeletal muscle substrate utilization

**Corresponding Author**

Elisabeth R. Barton, PhD

Applied Physiology & Kinesiology

College of Health & Human Performance

University of Florida

124 Florida Gym | 1864 Stadium Road

Gainesville, FL 32611 UNITED STATES

tel  352-294-1714 | fax 352-392-5262 | [erbarton@ufl.edu](mailto:erbarton@ufl.edu)

**Supporting Table ST1.** NMR parameters for different experiments

| **S.No.** | **Experiment** | **Spectrometer Frequency (MHz)** | **Spectral width (Hz)** | **Relaxation delay (d1)** | **Acquisition time (s)** | **Number of scans** | **Measuring Pulse (in degree)** | **Repetition time (s)** | **Acquiring Temperature (^o^C)** | **Decoupler/Presaturation** |
| --- | --- | --- | --- | --- | --- | --- | --- | --- | --- | --- |
| 1 | First slice of a NOESY pulse sequence | 600 MHz Bruker with TCl CryoProbe | 7211.54 | 1 | 4 | 64 | 90 | 5.2 | 25 | Presaturation pulse |
| 2 | ^13^C decoupling ON/OFF ^1^H experiment | 600 MHz Bruker with TCl CryoProbe | 7142.9 | 2.85 | 1.15 | 128 | 90 | 4.0 | 25 | WALTZ-16 |
| 3 | Conventional ^1^H decoupled ^13^C experiment | 600 MHz Agilent with 1.5 mmm superconducting (HTS) probe | 36764.7 | 1.5 | 1.5 | ~30K | 45 | 3.0 | 30 | WALTZ-16 |

**Supporting Table ST2.** Concentrations of branched chain amino acids, glycolytic, and TCA metabolites along with taurine and creatine in EDL muscle sample incubated in Ringer and MEM media (using [U- ^13^C_6_] glucose as a substrate), separately via 1D ^1^H NMR. Red = Branched chain amino acids (BCAA). “n” represents the biological replicates utilized in the study and each NMR sample consisted of pooled extracts of four individual muscles.

| Metabolites | EDL_ Ringer (mM) | EDL_MEM (mM) ±S.D. | Soleus_Ringer (mM) | Soleus_MEM (mM) ± S.D. |
| --- | --- | --- | --- | --- |
| Glutamate | 0.58 | 1.01±0.48 | 1.96 | 3.77±1.76 |
| Lactate | 35.41 | 49.84±27.24 | 15.81 | 25.26±20.15 |
| Alanine | 2.0 | 2.44±1.12 | 2.30 | 3.69±1.79 |
| Leucine | 0.10 | 1.40±1.02 | 0.12 | 1.42±1.01 |
| Isoleucine | 0.05 | 0.83±0.54 | 0.09 | 0.97±0.73 |
| Valine | 0.12 | 0.87±0.53 | 0.12 | 0.90±0.64 |
| Aspartate | 0.52 | 2.24±1.69 | 0.94 | 3.24±1.90 |
| Taurine | 48.19 | 56.86±24.08 | 44.45 | 62.46±29.56 |
| Creatine | 29.06 | 36.92±16.52 | 18.31 | 28.22±15.11 |
| Lactate/Alanine | 17.72 | 19.40±2.99 | 6.87 | 5.88±2.03 |
| Taurine/Creatine | 1.66 | 1.57±0.17 | 2.43 | 2.31±0.03 |

**Supporting Table ST3.** Fractional enrichment (%) ± S.D. in ringer and MEM media, in different skeletal muscle systems treated with either ^13^C_6_ glucose or ^13^C_3_ pyruvate. “n” represents the biological replicates utilized in the study and each NMR sample consisted of pooled extracts of four individual muscles.

|  | **Fractional Enrichment (%)** | | | | | | | |
| --- | --- | --- | --- | --- | --- | --- | --- | --- |
| **Media** | **MEM** | | | | **Ringers** | | | |
| **Substrate** | [U- ^13^C_6_] glucose | | [U-^13^C_3_] pyruvate | | [U-^13^C_6_] glucose | | [U-^13^C_3_] pyruvate | |
| **Metabolite** | Alanine (n=3) | Lactate (n=3) | Alanine (n=2) | Lactate (n=2) | Alanine (n=1) | Lactate (n=1) | Alanine (n=1) | Lactate(n=1) |
| **EDL** | 21.42 ± 10.82 | 6.01 ± 0.33 | 66.18 ± 7.22 | 14.73 ± 4.87 | 25.04 | 5.31 | 66.99 | 11.94 |
| **Soleus** | 27.62 ± 9.28 | 15.09 ± 1.25 | 61.81 ± 6.76 | 25.42 ± 4.17 | 27.70 | 17.67 | 67.46 | 24.37 |

**Supporting Table ST4.** Fraction of [1,2 ^13^C_2_] enriched acetyl-CoA (Fc3) and anaplerotic flux (C4/C3) values in ringer and MEM media, in different skeletal muscle systems treated with either ^13^C_6_ glucose or ^13^C_3_ pyruvate

|  | **MEM** | | **Ringers** | |
| --- | --- | --- | --- | --- |
| **Substrate & Muscle** | **Fc3 value** | **C4/C3** | **Fc3 value** | **C4/C3** |
| Glucose Soleus | NA | 2.59±0.10 | NA | 2.12 |
| Glucose EDL | NA | 1.43±0.19 | NA | 1.55 |
| Pyruvate Soleus | 0.39±0.03 | 1.36±0.24 | 0.44 | 0.97 |
| Pyruvate EDL | 0.30±0.02 | 0.83±0.02 | 0.76 | 1.04 |

**Supporting Table ST5.** Fraction of [1,2 ^13^C_2_] enriched acetyl-CoA (Fc3) and anaplerotic flux (C4/C3) values in different skeletal muscle systems in without and with pre-incubation with unlabeled pyruvate prior to labeled ^13^C_6_ glucose. “n” represents the biological replicates utilized in the study and each NMR sample consisted of pooled extracts of four individual muscles.

|  | No Pyruvate Pre-incubation | | Pyruvate Pre-incubation | |
| --- | --- | --- | --- | --- |
| Muscle | Enriched  acetyl-CoA  Fc3 (n=3) | Anaplerotic flux C4/C3 (n=3) | Enriched  acetyl-CoA  Fc3 (n=1) | Anaplerotic flux C4/C3 (n=1) |
| EDL | NA | 1.43±0.19 | NA | 3.602 |
| Sol. | NA | 2.59±0.10 | 0.245 | 4.066 |


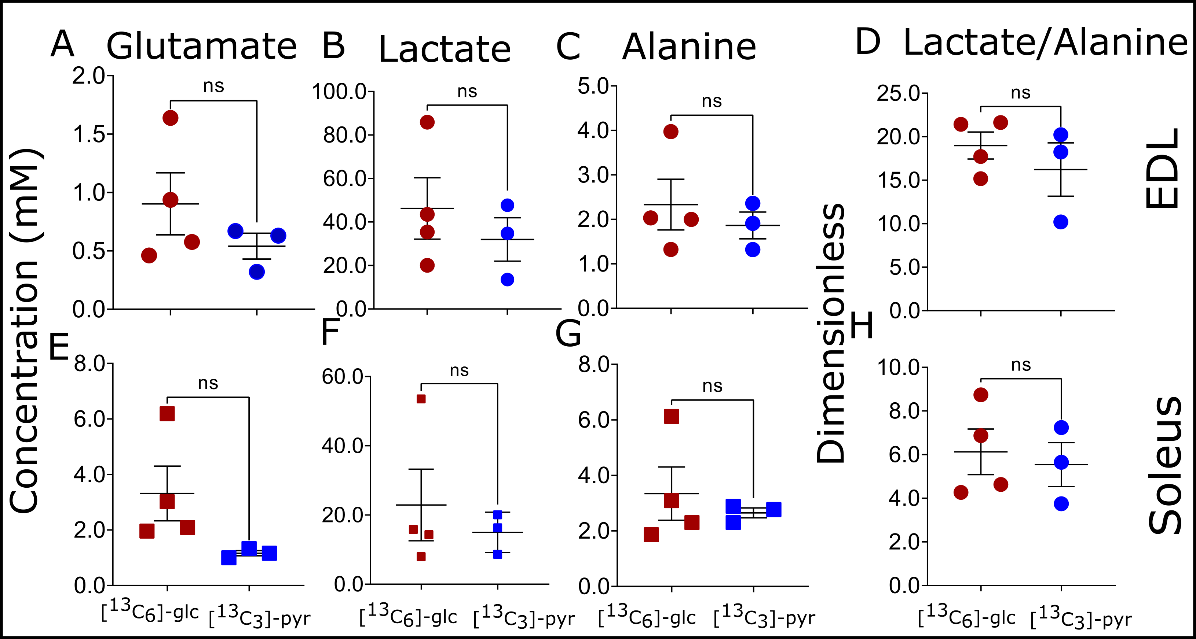


**Figure S1.** Comparison of concentrations of key TCA and glycolytic metabolites including lactate to alanine ratio in ^13^C labeled [U- ^13^C_6_] glucose and [U-^13^C_3_] pyruvate treated samples for isolated soleus and EDL muscles. Concentrations were determined using Chenomx NMR Suite 8.2 software, with ^13^C decoupling ON 1D ^1^H spectra. Each NMR sample is the extract of either four EDL or soleus muscles. Four biological replicates were used for [U- ^13^C_6_] glucose treated samples, and three biological replicates were used for [U-^13^C_3_] pyruvate treated samples. Each biological replicate was a pool of 4 individual muscles. Statistical significance was determined using unpaired t-tests via GraphPad Prism with p < 0.05 considered significant.


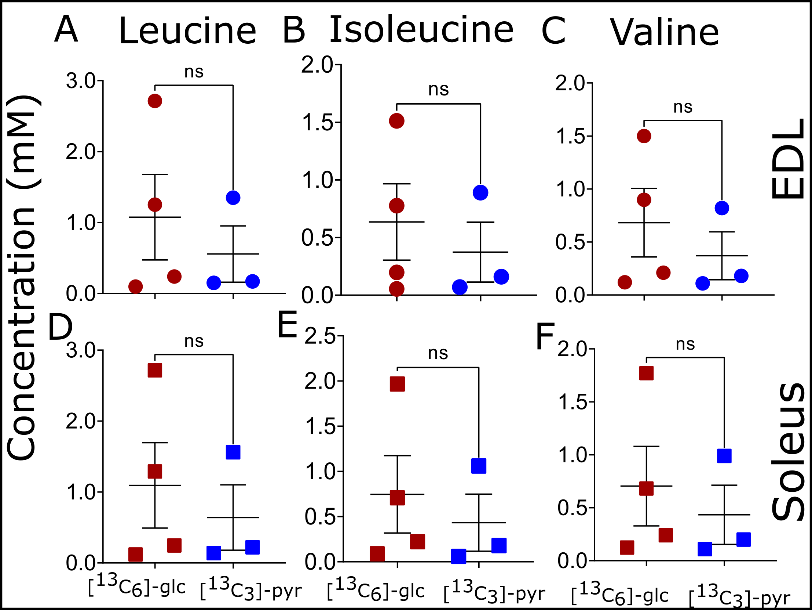


**Figure S2.** Comparison of concentrations of branched-chain amino acids in ^13^C labeled [U- ^13^C_6_] glucose and [U-^13^C_3_] pyruvate treated samples for isolated soleus and EDL muscles. Concentrations were determined using Chenomx NMR Suite 8.2 software, with ^13^C decoupling ON 1D ^1^H spectra. Each NMR sample is the extract of either four EDL or soleus muscles. Four biological replicates were used for [U- ^13^C_6_] glucose treated samples, and three biological replicates were used for [U-^13^C_3_] pyruvate treated samples. Each biological replicate was a pool of 4 individual muscles. Statistical significance was determined using unpaired t-tests via GraphPad Prism with p < 0.05 considered significant.


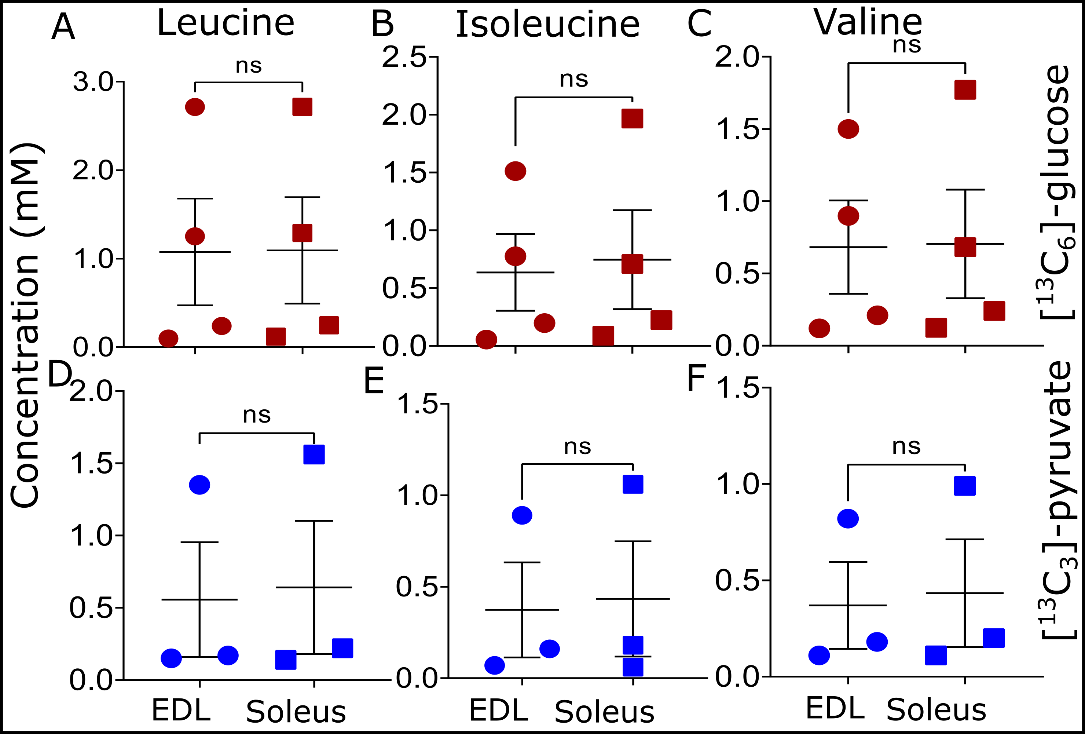


**Figure S3.** Comparison of concentrations of branched-chain amino acids in between soleus and EDL tissue samples incubated with ^13^C labeled [U- ^13^C_6_] glucose and [U-^13^C_3_] pyruvate. Concentrations were determined using Chenomx NMR Suite 8.2 software, with ^13^C decoupling ON 1D ^1^H spectra. Each NMR sample is the extract of either four EDL or soleus muscles. Four biological replicates were used for [U- ^13^C_6_] glucose treated samples, and three biological replicates were used for [U-^13^C_3_] pyruvate treated samples. Each biological replicate was a pool of 4 individual muscles. Statistical significance was determined using unpaired t-tests via GraphPad Prism with p < 0.05 considered significant.
